# Quality of sleep and health-related quality of life among health care professionals treating patients with coronavirus disease-19

**DOI:** 10.1192/j.eurpsy.2022.965

**Published:** 2022-09-01

**Authors:** J. Stojanov, A. Stojanov

**Affiliations:** 1 Special Hospital for Psychiatric Disorders, Admission Women’s Ward, Nis, Serbia; 2 Clinical Centre Nis, Clinic Of Neurology, Nis, Serbia

**Keywords:** Covid-19, Quality of sleep, health-related quality of life

## Abstract

**Introduction:**

Health care professionals exposed to coronavirus disease 2019 (COVID-19) are facing high levels of stress.

**Objectives:**

The aim was to evaluate the quality of sleep (QoS) and health-related quality of life (HRQoL), among health care professionals treating patients with COVID-19, as well as quantifying the magnitude of symptoms of depression and levels of anxiety.

**Methods:**

We included 201 health care professionals in a cross-sectional, web-based study by applying 7-item Generalized Anxiety Disorder (GAD-7) Scale, Zung Self-rating Depression Scale, 36-item Health Survey of the Medical Outcomes Study Short Form (SF36), Pittsburgh Sleep Quality Index (PSQI) and additional survey constructed for the purpose of the study.

**Results:**

Poor QoS and HRQoL correlated with high health anxiety and severe depressive symptoms and several demographic characteristics. Multiple linear regression analysis showed that higher scores on GAD-7 (beta = .71, p < .01) and lower scores on mental health (MH) subscale on SF36 questionnaire (beta = –.69; p < .01) were independent predictors of the higher PSQI score (adjusted R2 = .61, p < .01 for overall model). Higher scores on GAD-7 (beta = .68, p < .01) and worse self-perceived mental status (beta = .25; p < .05) were independent predictors of the lower SF36 scores (adjusted R2 = .73, p < .01 for overall model).

**Conclusions:**

The major MH burden of health care professionals treating infected patients during the COVID-19 pandemic indicates that they need psychological support.

**Disclosure:**

No significant relationships.

